# La Neuroplasticidad en el Trastorno de Estrés Postraumático

**DOI:** 10.31083/RN33478

**Published:** 2025-07-23

**Authors:** Beatriz López-López, Inmaculada Crespo

**Affiliations:** ^1^Departamento de Psicobiología, CES Cardenal Cisneros (Centro Adscrito a la Universidad Complutense de Madrid), E-28006 Madrid, España

**Keywords:** mielina, neuroplasticidad, psicodélicos, sinapsis, TEPT, trauma psicológico, myelin, neuroplasticity, psychedelics, psychological trauma, PTSD, synapses

## Abstract

**Introducción:**

El trastorno de estrés postraumático (TEPT) se desarrolla ante una experiencia traumática, real o amenazante, que produce emociones de miedo intenso y problemas de memoria, dañando significativamente la calidad de vida de las personas que lo manifiestan. En los últimos años comienzan a estudiarse los cambios anatomo-funcionales en el circuito amígdala-hipocampo-corteza prefrontal como factor clave tanto en la prevención, vulnerabilidad, y tratamiento del TEPT, siendo la neuroplasticidad uno de los factores con mayor interés. Por tanto, en esta revisión se abordarán los últimos datos publicados en relación con el TEPT y la neuroplasticidad.

**Desarrollo:**

Datos desde modelos preclínicos y clínicos apoyan que una experiencia traumática modifica tanto la plasticidad sináptica a través de variables electrofisiológicas y químicas, como la plasticidad mielínica que permite las conexiones a corta y larga distancia. Esta remodelación de los circuitos es clave de cara al desarrollo del TEPT. Sin embargo, también se asocia íntimamente con la prevención y con resultados positivos del tratamiento. Variables como el apoyo social o el uso de psicoterapia tras la vivencia de un trauma se relacionan con un buen pronóstico.

**Conclusiones:**

Se puede concluir que hay una interesante conexión entre la neuroplasticidad y el TEPT, aunque en la actualidad aún quedan abiertas muchas incógnitas y prometedoras líneas de prevención e intervención entre las que se incluyen las sustancias psicodélicas.

## Abstract (en)

**Introduction:**

Post-traumatic stress disorder (PTSD) develops in response to a traumatic experience, whether real or threatening, which produces emotions of intense fear and memory problems, significantly damaging the quality of life of those who manifest it. In recent years, anatomical-functional changes in the amygdala-hippocampus-prefrontal cortex circuit have begun to be studied as a key factor in the prevention, vulnerability, and treatment of PTSD, with neuroplasticity being one of the factors of greatest interest. Therefore, this review will address the latest published data regarding PTSD and neuroplasticity.

**Development:**

Data from preclinical and clinical models support that a traumatic experience modifies both synaptic plasticity through electrophysiological and chemical variables, as well as myelin plasticity which enables short and long-distance connections. This remodelling of circuitry is crucial for the development of PTSD. However, it is also closely associated with prevention and positive treatment outcomes. Variables such as social support or the use of psychotherapy following a traumatic experience are linked to a good prognosis.

**Conclusions:**

Therefore, there is an interesting connection between neuroplasticity and PTSD, although many questions remain open today, along with promising lines of prevention and intervention, including psychedelic substances.

## 1. Introducción

Las respuestas ante el miedo y el temor frente a la adversidad suponen un papel adaptativo y necesario para la supervivencia, aunque en ocasiones pueden tener efectos negativos sobre la calidad de vida de las personas [1]. Estas respuestas se manifiestan mediante reacciones fisiológicas corporales y cambios en el comportamiento. Sin embargo, las respuestas desmedidas pueden provocar un daño incapacitante, el trastorno de estrés postraumático (TEPT), definido por el manual diagnóstico y estadístico de los trastornos mentales texto revisado (DSM5-TR) por síntomas intrusivos, evitación, alteraciones emocionales y cognitivas y cambios en el *arousal* y la reactividad [2].

La prevalencia global a lo largo de la vida varía entre el 1–8% [3]. Si bien un alto porcentaje de los adultos se exponen a un tipo de estrés traumático, tan solo un 25–35% de estos desarrolla tal indefensión y un estrés duradero en el tiempo definiéndose en ese caso como postraumático y creando, en las situaciones más graves, un daño en las víctimas duradero y nocivo donde la memoria y el manejo emocional son las características centrales [4, 5].

La adquisición y consolidación de la memoria traumática autobiográfica, así como los mecanismos que regulan la extinción, se vinculan con el circuito que incluye a la corteza prefrontal (CPF), formación hipocampal y amígdala [6] y en el que el eje hormonal del estrés juega un papel relevante [7]. Este eje implica tres hormonas, la hormona liberadora de corticotropina (CRH), la hormona adrenocortoticotropina (ACTH) y el cortisol, esta última actuando como una señal de retroalimentación negativa a través de los receptores para glucocorticoides. El eje es modulado a lo largo de toda la vida por los mecanismos de neuroplasticidad, un proceso que, como respuesta al entorno externo e interno, permite la reorganización de las estructuras, de las conexiones y en último término del funcionamiento del Sistema nervioso central (SNC) [8].

Por tanto, el principal objetivo de esta revisión es analizar el TEPT desde la lente de la neuroplasticidad, no sólo la clásica plasticidad sináptica sino también la emergente plasticidad mielínica, para en último término analizar herramientas que potencien tanto la prevención como el mejor tratamiento individualizado del TEPT.

## 2. La Plasticidad Dependiente de la Experiencia en TEPT 

La plasticidad dependiente de la experiencia es un factor clave sustentado, entre otros, en la actividad neuronal y que ejerce un papel no solo en la formación de las sinapsis y el peso de las conexiones, sino también en la mielinización de los axones y el establecimiento de circuitos [9, 10].

Los cambios en la plasticidad sináptica se vinculan con el TEPT puesto que afectan a los distintos dominios conductuales asociados a este trastorno (Tabla 1, Ref. [5, 6, 8, 11, 12, 13, 14, 15, 16, 17]). En este sentido, el uso de un agente anestésico de acción corta en un modelo preclínico, mejoró el aprendizaje y memoria restaurando los problemas en plasticidad sináptica en el hipocampo [18]. Estos datos también se replicaron en personas diagnosticadas de TEPT [19] aunque en otro estudio no hubo asociación [20]. Además, el aprendizaje y la extinción de las amenazas, así como la evitación o el afrontamiento activo, se asocian a cambios estructurales y funcionales que implican a la CPF, la amígdala y la formación hipocampal [4]. Personas con TEPT demuestran una pérdida sináptica en la región dorsolateral de la CPF, una de las regiones encargadas de la regulación cognitiva [9].

**Tabla 1.** **Resumen de los principales dominios neurofisiológicos alterados y sus efectos en el TEPT**.

Dominio	Efectos	Referencias
Sueño	· Aumento de pesadillas y terrores nocturnos	[8, 12, 13]
	· Mayor latencia en el inicio del sueño	
	· Menor cantidad de sueño	
	· Sueño fragmentado	
	· Mayor número de movimientos oculares rápidos	
	· Ausencia de catatonía	
	· Aumento de la tasa cardíaca	
	· Aumento de alteraciones respiratorias	
Alerta/*Arousal*	· Sobreactivación y estado de alerta ante amenazas	[6, 8]
	· Aumentos en el reflejo acústico de sobresalto	
	· Irritabilidad	
	· Pánico	
	· Disrupción del sueño	
	· Estado de hipervigilancia	
	· Alteraciones cognitivas	
Memoria	· Recuerdos traumáticos e intrusivos	[5, 6, 11, 13, 14, 15, 16, 17]
	· Hipermnesia o amnesia disociativa	
	· Déficits en memoria declarativa	
	· Alteración del sistema de control mnésico: defectos en el control de los intentos de supresión de recuerdos	
	· Vivencia de los recuerdos traumáticos en el presente desconectados del tiempo, espacio y entorno, en general	

TEPT, trastorno de estrés postraumático.

**Fig. 1.**
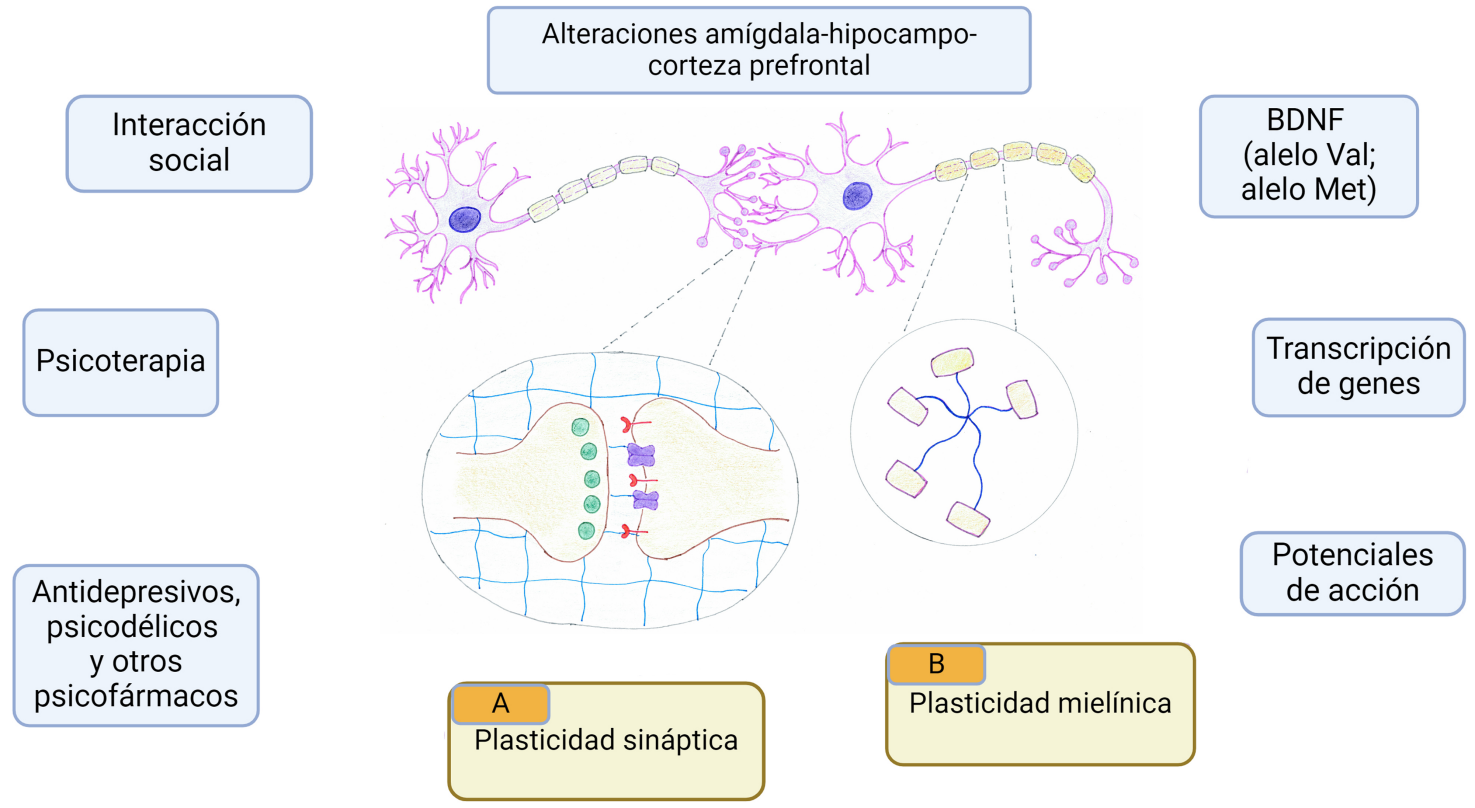
**Representación esquemática de los factores que modulan la neuroplasticidad en personas con TEPT**. Conjunto de factores y biomarcadores que interfieren en la neuroplasticidad y son claves en el desarrollo de TEPT. (A) Detalle de la plasticidad sináptica (verde BDNF, rojo oscuro receptor de BDNF TrkB, morado oscuro receptor AMPA y azul claro matriz extracelular). (B) Detalle de la plasticidad mielínica (morado claro oligodendrocito mielinizante). BDNF, factor neurotrófico derivado del cerebro; TrkB, receptor quinasa B de tropomiosina; AMPA, ácido α-amino-3-hidroxi-5-metil-4-isoxa- zolpropiónico.

Gracias al fortalecimiento de las sinapsis la memoria queda preservada, lo que favorece el funcionamiento cerebral de cada individuo. Entre los muchos factores reguladores, los electrofisiológicos ocupan un lugar destacado (Fig. 1). De esta forma, estudios recientes en humanos para modular la actividad eléctrica de la CPF ventromedial con estimulación magnética transcraneal en una muestra de 28 participantes [21] o la estimulación transcraneal de corriente continua en una muestra de 130 participantes con un procedimiento de evaluación ciego [22], mostraron mejoras en el recuerdo de la extinción a través del paradigma de miedo condicionado. Los resultados apuntan en el mismo sentido en personas con TEPT, aunque es necesario ampliar este enfoque [23].

Junto a los factores electrofisiológicos también conviene destacar el papel de los factores de crecimiento, pues se encargan de conservar la plasticidad neuronal [24]. En concreto, el factor neurotrófico derivado del cerebro (BDNF) es una proteína que participa en el mantenimiento de la plasticidad sináptica y la neurogénesis, apoyando la diferenciación, maduración y supervivencia neuronal [25, 26]. La señalización BDNF (Tabla 2, Ref. [11, 25, 26, 27, 28, 29]) ha estado implicada en el aprendizaje y extinción del miedo en sujetos con TEPT, especialmente en la CPF, hipocampo y amígdala [11, 26]. Igualmente, BDNF regula la expresión de CRH en el núcleo paraventricular del hipotálamo, lo que supone un vínculo con el estrés [27]. Así, aquellas alteraciones mentales relacionadas directa o indirectamente al estrés se asocian a BDNF, el cual puede ser una diana terapéutica interesante (Fig. 1A).

**Tabla 2.** **Papel de BDNF en el TEPT**.

Función	Efectos en TEPT	Referencias
Modula la plasticidad sináptica y la neurogénesis	Formación de circuitos cerebrales anómalos	[25, 26]
Influye en los procesos de aprendizaje y extinción del miedo	Cambios en los procesos de memoria	[11, 26]
Regula la expresión CRH	Vínculo con el estrés	[27]
Influye sobre la actividad del receptor glutamatérgico NMDA	Potenciación a largo plazo de recuerdos traumáticos	[28]
Regula los receptores glutamatérgicos AMPA	Altera la memoria a corto plazo y favorece la acción del receptor NMDA	[29]

CRH, hormona liberadora de corticotropina; NMDA, ácido N-metil-D-aspartato.

Se conoce que tanto BDNF (alelo Val) como su variante Val66Met (alelo Met) influyen en la aparición de comportamientos condicionados por la fobia y la vulnerabilidad, así como en la memoria contextual [24], lo cual funciona como un agravante del suceso traumático en el caso del alelo Met [11]. Es por esto por lo que este factor es un buen predictor del estrés, así como de otros comportamientos relacionados con el TEPT. Además, este marcador genético alelo Met correlaciona con la atrofia del hipocampo y la amígdala, lo cual indica la razón de vulnerabilidad a los rasgos neuróticos y trastornos del ánimo provocados por un aumento del nivel de activación o *arousal* [25, 30], aunque existen datos heterogéneos por variaciones metodológicas.

El alelo Met reduce la eficiencia de transcripción, plegamiento y transporte de BDNF, produciendo una reducción del tamaño y actividad del hipocampo, de la CPF y de la amígdala y afectando a la potenciación a largo plazo y a los procesos de extinción. Así, la presencia del alelo Met reduce la secreción de BDNF dependiente de la actividad, lo que se asocia a déficits en memoria episódica frente a sujetos con alelo Val [11]. Aquí cabría decir que el genotipo Val/Val es un factor protector mientras que el genotipo Val/Met o Met/Met sería un factor de riesgo para el TEPT. Por ello, se ha propuesto la hipótesis de la sensibilidad al estrés según la cual la interrupción de la actividad de BDNF, por ejemplo por la variante Met, favorece la aparición del estrés y otras alteraciones asociadas con el mismo [24].

Por último, es interesante analizar la relación de BDNF con los receptores glutamatérgicos ácido N-metil-D-aspartato (NMDA) y ácido α-amino-3-hidroxi-5-metil-4-isoxa- zolpropiónico (AMPA), implicados en la formación de nuevos recuerdos. BDNF puede aumentar la actividad del receptor NMDA [28], un receptor clave en la formación de recuerdos traumáticos y regular positivamente la expresión y localización de los receptores AMPA, así como su actividad [29], lo cual modula la plasticidad y excitabilidad neuronal. Al mismo tiempo la entrada de calcio a través de los receptores NMDA favorece la expresión de BDNF [31].

En esta misma línea, se ha observado que el uso de D-cicloserina, un agonista parcial de los receptores NMDA, mejora los síntomas del TEPT significativamente [32], aunque también se han encontrado diferencias no significativas con respecto al grupo placebo [33, 34].

Por tanto, la plasticidad sináptica es clave en todo este proceso, pero también debe ser considerada la plasticidad mielínica (Fig. 1B) por su papel sobre los circuitos neuronales en los procesos de aprendizaje y memoria [10]. Los oligodendrocitos, las principales células productoras de mielina, expresan receptores para glucocorticoides, lo que hace posible la asociación entre mielinización y estrés [35].

La resiliencia, entendida como la adaptación dinámica posterior a un trauma, es un factor determinante en la vulnerabilidad para desarrollar TEPT [5] que puede estar asociada a la mielinización [36]. Se ha descrito que la memoria de miedo contextual, al igual que otras memorias, requiere que se genere nueva mielina; por ello el uso de fármacos promielinizantes como el fumarato de clemastina en ratones mejora el recuerdo de memoria remota y favorece la generalización del miedo [37]. En veteranos de guerra con y sin TEPT, estimando el grado de mielinización a través de imagen de resonancia magnética ponderada en T1/T2, se ha observado que hay una correlación positiva entre el índice total de la escala de TEPT administrada por un médico (CAPS, por sus siglas en inglés) y la mielinización en el hipocampo. Además, esta misma correlación se observa respecto a la severidad de los síntomas depresivos [36].

En un estudio traslacional reciente centrado en el hipocampo, la amígdala y el cuerpo calloso se describen resultados similares en ratas y humanos [38]. En roedores, el análisis de la mielinización en la sustancia gris del giro dentado del hipocampo muestra una correlación positiva entre la densidad de los oligodendrocitos y la proteína básica de mielina, una proteína que se traduce solo en los oligodendrocitos maduros mielinizantes, y el fenotipo de evitación y ansiedad, mientras que en la amígdala y en las regiones cornu ammonis (CA) del hipocampo esta correlación positiva se produce con el aprendizaje del miedo contextual [38]. Esto corrobora los datos de un estudio previo usando esta misma proteína, junto a los oligodendrocitos específicos del giro dentado del hipocampo, que muestra que están asociados a la hipervigilancia, evitación y escape ante el estrés a partir de las dos semanas de su manifestación [39].

En cuanto a los humanos, al analizar las subescalas del CAPS los resultados indican que la mielinización en el hipocampo correlaciona positivamente con la evitación de lugar y el hiperarousal, mientras que la mielinización de la amígdala se asocia al distrés psicológico y físico, la evitación emocional y la respuesta de sobresalto [38].

Sin embargo, el estrés crónico percibido correlaciona de forma inversa con la mielina intracortical (ICM) del giro supramarginal derecho, implicada en la salud mental. Asimismo, esta ICM está negativamente correlacionada con el apoyo emocional centrado en el cuidado afectivo, pero no con el apoyo instrumental que incluye recursos para hacer frente a las dificultades [40]. Debido al papel del giro supramarginal derecho en la diferenciación afectiva del yo y del otro [41] es posible que individuos con un nivel más alto de ICM en esta estructura sean más empáticos y eviten juicios sociales sesgados, lo que les permitiría percibir un apoyo emocional más fuerte de otros en su red social cuando lidian con eventos estresantes [40].

Pero en el TEPT no sólo se altera la mielinización de la materia gris asociada a regiones restringidas donde están implicadas las interneuronas y neuronas excitatorias, sino que también se han descrito cambios en la mielinización de las conexiones a larga distancia que interconectan regiones alejadas, denominado habitualmente mielinización de la materia blanca [10].

Dianas analizadas a través de imágenes con tensor de difusión incluyen tractos frontolímbicos como el cíngulo, el cuerpo calloso, el fórnix o el uncinado, los cuales están alterados significativamente en personas con TEPT [42, 43, 44]. Además, en el estudio más amplio realizado hasta la fecha con 3047 participantes, se muestran alteraciones en la conectividad estructural en un tracto del cuerpo calloso denominado *tapetum*; este tracto conecta los lóbulos temporales, incluyendo el hipocampo derecho e izquierdo [45]. No obstante, en este estudio no es posible discernir si este efecto es un marcador de vulnerabilidad anterior a la aparición del TEPT o una respuesta patológica al trauma, ya que los datos utilizados son transversales; de igual forma hay que ser prudentes a la hora de establecer una relación de causalidad.

Así pues, tanto la plasticidad sináptica como la mielínica se asocian al desarrollo del aprendizaje, la flexibilidad y del bienestar humano, lo cual puede quedar suspendido a través de la exposición al estrés o al trauma. Esto abre la puerta a distintas intervenciones que permitan su modulación y por tanto una mejoría en la calidad de vida tras sufrir un evento traumático.

## 3. Estrategias de Prevención e Intervención Basadas en la Neuroplasticidad

El estrés percibido se puede definir como un marcador de la salud física y mental. Para ello, el afrontamiento del estrés requiere el empleo de ciertos recursos necesarios, como son el apoyo social, tanto emocional referido al cariño y al afecto, como instrumental asociado con la asistencia financiera y el tiempo de calidad con los demás [40], pudiendo estar ambos interrelacionados. En este punto cabría preguntarse por la base neurobiológica que se esconde detrás de estos efectos asociados con la interacción social, y una de las líneas más prometedoras de nuevo sería la neuroplasticidad [46]. La falta de apoyo social puede ser definida como un predictor de la aparición de alteraciones del estrés, puesto que esta está relacionada con una mayor sensibilidad hacia el mismo [1].

En este sentido, el restablecimiento del aprendizaje de refuerzo social está acompañado de la restauración de la metaplasticidad, un término acuñado en 1996 sobre la regulación dinámica del grado en que se puede inducir plasticidad sináptica [47]. Para ello, la oxitocina se perfila como un factor crítico dentro del núcleo *accumbens *donde la expresión de genes de remodelación de la matriz extracelular permite que dendritas y axones queden libres y puedan formar nuevas conexiones (Fig. 1A) [48].

Junto al papel de la interacción social y la metaplasticidad que facilita que las neuronas sean más sensibles a estímulos que inducen plasticidad, el proceso de neuroplasticidad puede modificarse también a través de factores ambientales como la psicoterapia o el uso de fármacos. Es por ello por lo que la Guía de práctica clínica de *Veteran Affairs/Department of Defense* (VA/DoD) de 2023 aconseja el uso de psicoterapias centradas en el trauma como primera línea de intervención en el TEPT y si la psicoterapia no está disponible recomienda la farmacología [49].

Las psicoterapias individuales más recomendadas para el tratamiento del TEPT incluyen la terapia de procesamiento cognitivo, la desensibilización y reprocesamiento del movimiento ocular (EMDR), o la exposición prolongada [49]. La primera es un tipo de terapia cognitivo conductual (TCC) donde se enseña cómo evaluar y cambiar los pensamientos perturbadores que ha tenido desde su trauma [50]. Se ha descrito que el comienzo temprano en la intervención con TCC es necesario, como medida de prevención secundaria y potencialmente eficaz, para que los resultados alcanzados se mantengan a largo plazo [51].

El uso de la terapia de procesamiento cognitivo y la mejoría de los síntomas en personas con TEPT se ha asociado con cambios en la sustancia blanca en regiones como la cápsula interna izquierda, la rama posterior de la cápsula interna, la circunvolución del cíngulo izquierdo, el fascículo longitudinal superior y el esplenio del cuerpo calloso [52]. De igual forma, el tratamiento con TCC o EMDR se asocia a cambios en la microestructura de la sustancia blanca del cíngulo dorsal, el principal tracto de materia blanca que conecta la corteza cingulada anterior, en personas con TEPT en remisión y TEPT persistente; así, mientras en los pacientes con TEPT persistente aumenta la anisotropía fraccional (AF), un índice de la integridad de la sustancia blanca, después del tratamiento en los pacientes en remisión y en los controles no se observa este incremento [53]. Estos autores hipotetizan que se puede estar dando un aprendizaje de miedo a través de la hiperactividad de la corteza cingulada durante las intrusiones en los pacientes resistentes, lo que se asociaría a la mayor mielinización del haz del cíngulo dorsal [53]. Sin embargo, una explicación alternativa no excluyente podría estar en la implicación de la corteza cingulada anterior en el aprendizaje de la extinción y la regulación de las emociones [4].

Otro estudio interesante muestra que el uso de TCC basada en el trauma reduce la AF de la sustancia blanca y correlaciona con la mejoría de los síntomas de disforia que incluyen factores como la evitación pasiva, alteraciones del sueño, dificultades de concentración e irritabilidad. Entre las regiones incluidas en este estudio cabe destacar regiones límbicas, haces interhemisféricos, fibras de proyección, fibras de asociación y tractos del tronco del encéfalo [54].

En ocasiones no es posible o suficiente el uso de psicoterapia, por lo que habría que acudir a la psicofarmacología. Actualmente la opción más común para el tratamiento del TEPT es el uso de dos inhibidores selectivos de la recaptación se serotonina, sertralina y paroxetina, aunque es frecuente que no alcancen los resultados deseados [55]. Por ese motivo, se están buscando nuevas dianas y hoy existe un resurgir del uso de psicodélicos. Este grupo heterogéneo de sustancias incluye moléculas que alteran los estados perceptivos, como la ibogaína o la psilocibina, que favorecen la conexión emocional, como el 3-4 metilenedioximetanfetamina (MDMA), o que inducen efectos disociativos, como la ketamina. Todos ellos tienen en común su capacidad para modular la neuroplasticidad e influir sobre los circuitos neuronales [56]. Sin embargo, la evidencia para recomendar a favor o en contra del uso de psicodélicos es insuficiente [49].

Analizando tres de ellos, se puede observar que el tratamiento con ibogaína, un psicodélico atípico coadministrado con magnesio, produce mejoras en TEPT, en depresión y en ansiedad (Ensayo clínico registrado: NCT04313712 https://clinicaltrials.gov/study/NCT04313712) [57]. Estos efectos fueron inmediatos y se mantuvieron un mes después del tratamiento en personas de las fuerzas especiales y veteranos de guerra con TEPT sin causar alucinaciones o paradas cardíacas, pero aún no está legalizado su utilización como fármaco [57]. Los resultados de este estudio, aunque son preliminares y prometedores, requieren ensayos controlados aleatorios adicionales con muestras más grandes.

En cuanto al MDMA, este entactógeno ha sido rechazado recientemente por la *Food and Drug Administration* (FDA) a partir de dos ensayos clínicos [58, 59] y las indicaciones de un comité asesor por falta de datos. La idea subyacente del uso de esta droga no está tanto en el tratamiento en sí y la remisión de los síntomas por el fármaco, sino en la implicación que tiene de cara a la apertura terapéutica a los eventos traumáticos.

Finalmente, la ketamina, un antagonista no competitivo del receptor de glutamato NMDA, puede resultar eficaz al disminuir los síntomas de TEPT [59]. La administración de esta droga bloquea la vía receptora de NMDA, un receptor asociado a la formación de nuevas conexiones y modula la transcripción de proteínas de la membrana extracelular [48]. Se cree que el bloqueo de los receptores NMDA media la activación del receptor AMPA en las interneuronas GABA y la desinhibición de la señalización del glutamato [60]. Además, tanto la ketamina como otros psicodélicos se unen al receptor BDNF, asociado al crecimiento neuronal, favoreciendo las conexiones y la plasticidad cerebral gracias a la modulación de las conexiones sinápticas [56, 61]. Además, en el análisis de la densidad sináptica en humanos a través de tomografía por emisión de positrones, usando un radioligando que se une a la proteína presináptica (SV2A), se vio que, a pesar de la reducción de síntomas en pacientes con TEPT, no hubo cambio en la densidad sináptica; sin embargo, los análisis *post-hoc* sugirieron que los efectos pueden estar en la restauración de la sinapsis en pacientes que presentan como línea base bajos niveles de SV2A [62].

En la actualidad, el uso de Ketamina no se recomienda para el tratamiento del TEPT aislado, dado que el conjunto de pruebas tiene limitaciones, incluida la falta de pruebas de eficacia, los riesgos y efectos secundarios de la ketamina. Sin embargo, teniendo en cuenta la alta comorbilidad del TEPT con el trastorno depresivo mayor, en estos casos sí que se podría valorar la ketamina o la esketamina (Spravato) para tratar la depresión en pacientes con ambas afecciones [49]. Asimismo, están en el foco el desarrollo de nuevos fármacos similares a la ketamina con acciones rápidas y sin los efectos secundarios [63].

Los efectos que los psicodélicos, administrados en entornos seguros, tienen sobre la neuroplasticidad pueden facilitar una mejora en la psicoterapia haciendo que el efecto terapéutico sea más que la suma de sus partes [64]. Sin embargo, son necesarios más estudios que analicen esta relación. De esta forma, cuanto mejor se conozcan los efectos que tienen (o no tienen) las distintas estrategias de intervención en el TEPT, más fácil será generalizar los resultados y ofrecer a las personas alternativas para mejorar su calidad de vida.

## 4. Conclusiones

Los cambios anteriormente descritos nos llevan a la idea de que tanto la formación/remodelación de circuitos como la estabilidad de los mismos, se traduce en papeles cruciales para la prevención o el diagnóstico del TEPT, y para el proceso terapéutico. Así pues, a través de la desconexión de los recuerdos emocionales y reconfiguración posterior, usando para ello la interacción social, la psicoterapia e incluso agentes psicodélicos, se vislumbraría un futuro más esperanzador para estas personas. Sin embargo, este camino es exigente dada la naturaleza heterogénea del TEPT, haciendo necesario superar dificultades metodológicas como son el uso de muestras pequeñas y de diseños transversales o la dificultad para establecer causalidad, lo que supone un reto motivador para la comunidad científica. En este sentido, nuevas líneas de estudio centradas por ejemplo en el seguimiento a largo plazo de las personas antes, durante y después de manifestar TEPT, en intervenciones preventivas basadas en la plasticidad cerebral o en el papel de factores culturales sobre aspectos neurofuncionales, se vislumbran de forma prometedora de cara a la comprensión de este trastorno tan devastador.

## Contribuciones de los Autores

BLL e IC adquirieron y analizaron la información; BLL e IC redactaron los distintos apartados del artículo; IC diseñó la revisión e hizo la revisión crítica del manuscrito. Ambos autores contribuyeron a los cambios editoriales del manuscrito. Ambos leyeron y aprobaron el manuscrito final. Ambos autores participaron adecuadamente en el trabajo y aceptaron ser responsables de todos sus aspectos.

## Aprobación Ética y Consentimiento Informado

No aplicable.

## Agradecimientos

Agradecemos la ayuda recibida por parte de Ana Domínguez Sahagún en el proceso de elaboración de la figura 1. También agradecemos el soporte recibido por parte del CES Cardenal Cisneros.

## Financiación

Esta investigación no recibió financiación externa.
